# Can complex programs be sustained? A mixed methods sustainability evaluation of a national infant and young child feeding program in Bangladesh and Vietnam

**DOI:** 10.1186/s12889-020-09438-2

**Published:** 2020-09-04

**Authors:** Corrina Moucheraud, Haribondhu Sarma, Tran Thi Thu Ha, Tahmeed Ahmed, Adrienne Epstein, Jeffrey Glenn, Hoang Hong Hanh, Tran Thi Thu Huong, Sharmin Khan Luies, Aninda Nishat Moitry, Doan Phuong Nhung, Denise Diaz Payán, Mahfuzur Rahman, Md Tariqujjaman, Tran Thi Thuy, Tran Tuan, Thomas J. Bossert, Margaret E. Kruk

**Affiliations:** 1 University of California Los Angeles, Fielding School of Public Health, 650 Charles E. Young Dr. S, Los Angeles, CA 90095 USA; 2 icddr,b, Nutrition and Clinical Services Division, Dhaka, Bangladesh; 3 Research and Training Centre for Community Development (RTCCD), Hanoi, Vietnam; 4 Harvard T.H. Chan School of Public Health, Boston, MA USA; 5 Present Address: University of California San Francisco School of Medicine, San Francisco, USA; 6 Present Address: Brigham Young University College of Life Sciences, Provo, USA; 7 Present Address: University of California Merced, Department of Public Health, San Francisco, USA

**Keywords:** Sustainability, Mixed methods, Global health

## Abstract

**Background:**

Poor early-life nutrition is a major barrier to good health and cognitive development, and is a global health priority. Alive & Thrive (A&T) was a multi-pronged initiative to improve infant and young child feeding behaviors. It aimed to achieve at-scale child health and nutrition improvements via a comprehensive approach that included nutrition counseling by health workers, policy change, social mobilization and mass media activities. This study evaluated the sustainability of activities introduced during A&T implementation (2009–2014) in Bangladesh and Vietnam.

**Methods:**

This was a mixed methods study that used a quasi-experimental design. Quantitative data (surveys with 668 health workers, and 269 service observations) were collected in 2017; and analysis compared outcomes (primarily dose and fidelity of activities, and capacity) in former A&T intervention areas versus areas that did not receive the full A&T intervention. Additionally, we conducted interviews and focus groups with 218 stakeholders to explore their impressions about the determinants of sustainability, based on a multi-level conceptual framework.

**Results:**

After program conclusion, stakeholders perceive declines in mass media campaigns, policy and advocacy activities, and social mobilization activities – but counseling activities were institutionalized and continued in both countries. Quantitative data show a persisting modest intervention effect: health workers in intervention areas had significantly higher child feeding knowledge, and in Bangladesh greater self-efficacy and job satisfaction, compared to their counterparts who did not receive the full package of A&T activities. While elements of the program were integrated into routine services, stakeholders noted dilution of the program focus due to competing priorities. Qualitative data suggest that some elements, such as training, monitoring, and evaluation, which were seen as essential to A&T’s success, have declined in frequency, quality, coverage, or were eliminated altogether.

**Conclusions:**

The inclusion of multiple activities in A&T and efforts to integrate the program into existing institutions were seen as crucial to its success but also made it difficult to sustain, particularly given unstable financial support and human resource constraints. Future complex programs should carefully plan for institutionalization in advance of the program by cultivating champions across the health system, and designing unique and complementary roles for all stakeholders including donors.

## Background

The first thousand days of life are critical for child nutrition, growth and health outcomes [1–4] – yet breastfeeding and complementary feeding practices remain suboptimal in many low- and middle-income countries [4, 5], and numerous infant and young child feeding (IYCF) interventions have struggled to achieve impact at-scale [6, 7]. A noteworthy exception is the Alive & Thrive initiative (A&T), which was implemented in Bangladesh, Ethiopia and Vietnam between 2009 and 2014 with financing from the Bill and Melinda Gates Foundation [8]. Evidence indicates that A&T improved IYCF behaviors and outcomes [9–11] -- but little is known about whether the program’s activities and effectiveness persisted beyond the end of the project period, nor what factors contributed to sustained activities or outcomes.

Sustainability is a critical issue: after a period of rapid growth, global health development assistance has slowed and is showing signs of stagnation if not decline [12]. Scholars, policymakers and practitioners are interested in what factors enable the continuation of externally-funded programs after the initial project period ends [13–21]. International organizations and donors are increasingly attentive to “graduation” (i.e., how to transition responsibilities) as funding is phased out [22–24]. Despite active dialogue, there is little rigorous empirical evidence on the drivers of health program sustainability particularly from low-resource settings [25, 26]. Additionally, the concept of sustainability has evolved for several decades yet there remains little consensus about how to define or measure it [21, 27–29].

This study aims to add to this literature by using a theory-driven empirical approach to evaluate the sustainability of activities implemented during the A&T project period (2009–2014) in Bangladesh and Vietnam, by asking: (1) To what extent were *activities* undertaken during A&T ongoing in Bangladesh and Vietnam, 2 years after the end of external program funding? (2) What were stakeholders’ impressions about *determinants* of the sustainability of the A&T model and activities?

## Methods

### Overview of A&T in Bangladesh and Vietnam

The overall objective of A&T in Bangladesh and Vietnam was to demonstrate a large-scale model for achieving IYCF improvements in the participating countries. The exact A&T interventions in each country differed based on extensive formative research and dialogue with partners, but were informed by a common implementation framework and included four main components (and specific activities within each): advocacy, interpersonal communication and community mobilization, mass communication, and strategic use of data [30, 31]. Table 1 provides an overview of these components and activities therein; substantially more detail can be found in previous publications [8, 32–35]. The program was implemented with subnational variation: only certain geographic areas in each country received the A&T interpersonal counseling (delivered via household visits from health workers in Bangladesh, and via facility-based social franchises in Vietnam called Mặt Trời Bé Thơ, MTBT) and community mobilization activities, while mass media and policy/advocacy activities were implemented nation-wide. Health workers in the intervention areas were trained on IYCF counseling, and given resources (including supportive supervision and ongoing monitoring) to enable high-quality IYCF counseling activities.

**Table 1:** Overview of A&T activities

Intervention component	Bangladesh	Vietnam
**Interpersonal counseling**	IYCF counseling provided at household level by BRAC health workers (community-based volunteers: Shasthya Shebikas [SS] and Pushti Shebikas [PS]; and health workers: Shasthya Kormis [SK] and Pushti Kormis [PK])	Group and individual IYCF counseling at franchise locations within government health system, called Mặt Trời Bé Thơ (MTBT, “Little Sun”); IYCF community support groups (in remote locations)
**Mass media**	IYCF television ads, radio programs, community dialogue, quiz shows	IYCF television ads, signs, loudspeaker announcements, website and mobile phone app
**Community mobilization**	BRAC managers led meetings with local stakeholders; village theater shows on IYCF	Village health workers made household visits to invite families to counseling sessions
**Policy advocacy**	National IYCF Alliance established; workshops with technical stakeholders to disseminate IYCF data; engagement with journalists	National Nutrition Strategy and IYCF Action Plan established; technical support to strengthen national laws/codes
**Partnerships & alliances** **Strategic use of data**		

*IYCF: Infant and young child feeding*

*MTBT: Mặt Trời Bé Thơ (“Little Sun”)*

*SS: Shasthya Shebika*

*PS: Pushti Shebika*

*SK: Shasthya Kormi*

*PK: Pushti Kormi*

Sustainability strategies were a key aspect of A&T program design [33, 35]. These detailed which program elements would be most amenable to continuation after the official project period ended, and what resources would be needed in order to sustain their implementation by local partners. Examples included strengthening the policy environment through advocacy, identifying sources for financial support, encouraging adoption of key A&T activities by other implementing partners, disseminating A&T-developed tools to help build capacity, and stimulating demand for services through increased community awareness; for more details and examples from countries see Appendix 1.

### Framework

A recent review identified five different types of definitions of sustainability: those that focus on continuation of activities, on continuation of benefits, on capacity building, on adaptations, and on cost recovery [28]. Some definitions combine these components; e.g., Moore et al. posit that sustainability is a program, intervention or behavior change that continues, with or without adaptations, and produces benefits over time [29]. Other definitions specify a distinct concept of “sustainment” as the continuation of structures or processes [25] while others view “sustainment” as being about health outcome indicators, versus “sustainability” which is about capacity to continue a program or activities [36]. Still others view the process itself as what defines sustainability [19], or conceptualize sustainability as a complex, non-linear and dynamic system [16, 21, 37].

We take a broad view of sustainability as including activities, structures/processes, stakeholders, and capacity. Table 2 shows how we define, operationalize and measure sustainability in the context of this study. *Determinants of sustainability* are factors that influence the continuation of program services beyond the initial period. We posit that these determinants – sometimes also called “capacity for sustainability” [38] – are multilevel influences [19, 21, 39, 40] across program, organization, and context. Our analysis is informed by a seminal framework of sustainability determinants that originated in global health studies during the 1980s; the Appendix shows how this aligns with other common frameworks from the literature (Appendix 2).

**Table 2:** Conceptual framework for sustainability evaluation and operationalization of key variables

Domain	Constructs	Operationalization	Data source
Determinants of sustainability	Program/project-specific factors	● Clear goals ● Perceived effectiveness ● Financing ● Training ● Evaluation/assessment ● Leadership	● Interviews and focus groups ○ Bangladesh: 24 interviews, 43 focus group participants (7 groups) ○ Vietnam: 121 interviews, 30 focus group participants (6 groups)
	Organizational factors	● Local-level modifiability ● Donor/Initiative- client/−community interactions ● Project champion ● Integration with institutions ● Institutional strength/capacities	
	Contextual factors	● Concurrent projects/activities ● Community characteristics ● Political, economic and cultural characteristics	
Outcomes of sustainability	Continuation of program activities	● Dose and reach of program ● Adherence to program standards/guidelines	● Interviews and focus groups ○ Bangladesh: 24 interviews, 43 focus group participants (7 groups) ○ Vietnam: 121 interviews, 30 focus group participants (6 groups) ● Service observation ○ Bangladesh: 242 observations ○ Vietnam: 58 observations
	Capacity	● Knowledge ● Self-efficacy, job satisfaction	● Health worker surveys ○ Bangladesh: 600 surveys ○ Vietnam: 68 surveys

*Outcomes of sustainability* may find analogs in the broader implementation science literature: what is the continued dose, reach, and fidelity of activities over time, and what adaptations have occurred? Here we focus on dose and fidelity, i.e. which activities continued, and how consistent were these with the original implementation plan; and we examine the relationship with health worker capacity to implement.

We conceptualize sustainability determinants and outcomes as distinct, but related and equally critical, constructs [41]: there may be a deterioration of activities, which can influence health worker capacity if the system has not found ways to foster sustainability (through determinants). This deterioration may ultimately affect worker and caregiver behaviors and health outcomes, although these are beyond the scope of this analysis.

### Study setting and design

We collected data in Bangladesh and Vietnam, from areas that had received the full set of program activities (intervention areas), and areas that had not received interpersonal communication nor community mobilization activities (comparison areas). This allows a comparison of outcomes between intervention and comparison areas, in a quasi-experimental design in order to isolate the specific effect that may be attributable to A&T. The evaluation was led by an international team that was not involved with implementing or evaluating the original A&T project. Although Ethiopia participated in A&T during this time, it was not included in this evaluation because it received additional donor support to continue activities, beginning in 2014. A mixed methods study design allowed us to more thoroughly understand both the outcomes and the determinants of sustainability, as well as how these factors are interconnected in each country.

### Data instruments

There were two quantitative data instruments: health worker surveys and service observation checklists; these were informed by previous instruments [42] including from A&T evaluations [8, 31, 34, 43] and from previous surveys aimed at assessing health worker satisfaction and self-efficacy [44]. The main quantitative outcomes were: health worker IYCF knowledge, job satisfaction and self-efficacy (assessed by survey); and number of recommended messages/activities delivered during an IYCF counseling session (assessed by direct observation).

There were two qualitative data collection instruments, a semi-structured key informant interview guide and a focus group guide; which were developed based on a previous sustainability study [17]. The interview guides included questions about stakeholders’ perspectives of program successes, implementation facilitators and challenges, adaptations, replication, leadership capacity, existing partnerships, service quality, funding sources, monitoring and evaluation processes, and best practices. The focus group guide collected health worker supervisors’ views on the continuation of activities post-implementation, adaptations, sources of funding, integration with other programs, replication, implementation challenges and facilitators, human resources training and retention, and quality of services.

All instruments were developed first in English and revised with input from each country’s research team. The finalized version was translated into the local primary language of each country and verified using a back-translation process.

### Site and participant selection

#### Quantitative

In Vietnam, 3 of 15 provinces that participated in A&T were selected for quantitative data collection. These were purposively selected to overlap with the provinces that participated in the endline evaluation, and to represent variation in geographic region and in A&T program implementation quality (following discussions with the A&T program). Within these provinces, districts were stratified by whether they had MTBT franchises, and 12 were randomly selected (6 intervention and 6 comparison); within these, up to 3 communes per district were randomly selected (20 communes total). Random sampling of administrative units was conducted using a random number generator. Health workers in sampled areas were recruited from hospitals and commune clinics. All eligible workers – defined as those actively involved in client counseling, and, in intervention areas, who had worked at the health facility during A&T and were exposed to program training – were approached and invited to participate in the study.

In Bangladesh, the 5 divisions and 10 districts that participated in the A&T endline evaluation were all included here, including the same 20 sub-districts (upazilas) (10 intervention and 10 comparison) as participated in that evaluation. Health workers were identified by first randomly selecting 12 higher-level health workers (Shasthya Kormi/Pushti Kormi) from the roster in selected study areas, and then randomly selecting up to 9 health worker volunteers (Shasthya Shebika/Pushti Shebika) who they supervise. Random selection of health workers from these rosters was conducted using a random number generator. Members of the research team approached selected health workers and invited them to participate in the study. Eligible health worker volunteers had a planned household visit within two days of initial contact by the study team. In both countries, IYCF counseling sessions by the sampled health workers (to pregnant women or caregivers of a child < 24 months) on the day of data collection were observed, if caregivers consented.

The full sample selection procedure is illustrated in Appendix 3.

#### Qualitative

Key informant interviews (KII) were conducted with a purposive sample of national and sub-national stakeholders in both countries, including policymakers, IYCF experts, and representatives from non-governmental organizations, donors, development partners, and health professionals. Potential participants were identified via discussion with the A&T program and government partners, on the basis of their knowledge about A&T and IYCF activities subsequent to its conclusion. Recruitment stopped once the research team reported reaching data saturation for key questions. Focus group discussions (FGD) were conducted with supervisors and managers of health workers who deliver IYCF services, in intervention areas. Focus group participants were identified based on the sampling frame for quantitative data collection in both countries.

### Data collection

#### Quantitative

Data were collected between January–May 2017, by experienced data collectors who received specific training about this evaluation. After providing informed consent, health workers participated in the survey in a private location (average duration: 48 min, standard deviation 34); data were collected on Samsung tablets using SurveyCTO, and the encrypted data were uploaded to a server where they underwent routine quality checks. Experienced research staff (not involved in data collection) conducted quality assurance audits in the field during pilot data collection periods, and provided individual and overall feedback.

#### Qualitative

Trained research staff conducted the interviews and focus groups in the local language (Bengali and Vietnamese) in semi-private or private settings (e.g., office, conference room, etc.) where the individual/s worked, to promote comfort and privacy. All interviews and groups were audio recorded with permission from participants. There were four participants in Bangladesh who did not consent to audio record their interviews, so interviewers took detailed notes with quotes. In total, 24 interviews were conducted in Bangladesh (range: 30–90 min) and 121 interviews in Vietnam (range: 45–90 min). Seven groups were conducted in Bangladesh (6–7 participants each) which each lasted from 100 to 160 min in duration and six in Vietnam (4–5 participants each) which each lasted about 60 min in duration. Teams held daily debriefing discussions to discuss challenges and preliminary observations.

### Data analysis

#### Quantitative

All analyses were conducted in Stata v14.2. Knowledge, self-efficacy and job satisfaction scores were standardized to a 100-point scale; as were the service observation scores (which summed the number of tasks performed by the health worker during an IYCF counseling session). Analysis examined differences in these variables between intervention and comparison areas (α = 0.05), including with t-tests, chi-squared tests, and multivariable models. Models for health worker outcomes included geographic fixed effects (province in Vietnam, division in Bangladesh) and robust standard errors, as well as covariates of worker age, years of experience and years of education.

#### Qualitative

Audio recordings were transcribed verbatim, and the transcripts were imported to NVivo. A preliminary codebook was based on the study’s theoretical framework (with primary and sub-category codes reflective of constructs in Table 2). Each country team completed pilot coding on a subset of interviews, and the entire research team discussed these results to address discrepancies and revise code definitions for clarity. Country-specific emergent codes were also identified, discussed, and added to the codebook. For example, there is an electronic reporting system used for IYCF data in one country therefore a specific code was added to ensure content about this reporting system was captured. During these steps of codebook development and refinement, investigators from all teams (Bangladesh, Vietnam, USA) met regularly both in-person and by telephone, to discuss the process and agree on the final version of the codebook.

Two researchers from each country team coded the transcripts. Coders met regularly to iteratively review discrepancies, discuss results, and develop thematic summaries. Analyses included examining themes and areas of variation within each country’s qualitative data on determinants of sustainability, as well as identification of linkages between sustainability determinants and dimensions. Analytical summaries and exemplary quotes were translated into English by bilingual research staff. Since all qualitative data were collected in the local language, this data analysis process was conducted independently in each study country.

### Ethical review

This study was reviewed and approved by Institutional Review Boards at the Harvard T.H. Chan School of Public Health (protocol #16–1706), the University of California Los Angeles (protocol #16–001754), the Hanoi School of Public Health (Ethical Review Board for Biomedical Research, protocol #016–335) in Vietnam, and icddr,b (Ethical Review Committee, protocol #PR-16060) in Bangladesh. In Vietnam, an additional scientific protocol review was conducted by the Scientific Review Board of the Institute of Preventive Medicine and Public Health and Hanoi Medical University. All participants were at least 18 years of age and provided written consent to participate in the study.

## Results

Descriptive information on study participants can be found in Appendix 4.

### To what extent were *activities* undertaken during A&T ongoing in Bangladesh and Vietnam, 2 years after the end of external program funding?

In both study countries, some A&T activities were continued via institutionalization after the end of A&T. In Bangladesh, the government incorporated IYCF counseling into its health programs via the Institute of Public Health Nutrition; activities including household visits, growth monitoring, and community-based support groups, are implemented by staff from the Directorate General of Health Services and Directorate General of Family Planning of Ministry of Health and Family Welfare. In Vietnam, several provincial governments scaled up the MTBT franchises, and the government added IYCF counseling to the standard package of postnatal services. Policy and advocacy activities have continued in both countries, with coordination and support from the National IYCF Alliance in Bangladesh (established during the A&T project period) and the National Institute of Nutrition in Vietnam. Although both countries have lessened their mass media campaigns, some television advertisements have continued in certain geographies and/or on select occasions.

Other activities were substantially curtailed since the end A&T, including the elimination of entire program components and associated activities in Vietnam (social mobilization activities and community groups); and certain core activities, such as monitoring and evaluation, were also reduced in both countries.

*“Since the project ended, we stopped reporting because no one asked us to report. I asked Ms. X from commune Y, and she said there was no need to report so I stopped and no one has reprimanded me.”* (Vietnam, female, sub-national KII)

Interviewees noted cutbacks in counseling frequency due to budget reductions, and particularly mentioned limited or a lack of incentives for health workers to conduct IYCF counseling; competing priorities, especially activities that are income-generating; and waning interest from caregivers in Vietnam.

“*[Workers] have started saying that they will sell micronutrient powder rather than demonstrate IYCF … When I ask the reason, they mentioned that they got 200 taka incentives for [IYCF] activities before, but now they get nothing*” (Bangladesh, male, FGD)

*“It is practical to be demotivated if you are not receiving any money for work. The [workers] get incentives from various other programs including tuberculosis DOTS [directly observed treatment, short-course], ANC and PNC services, etc.” (*Bangladesh, male, sub-national, KII)

“*In the past, mothers came easily. The program was new and attractive, and they got a gift after the session. Now there is no gift, and it is no longer a new program, so it is somewhat difficult”* (Vietnam, male, sub-national KII)

Strong IYCF training was widely identified as a major achievement of A&T – but training efforts have substantially decreased, in terms of frequency, type (especially refresher trainings) and mechanisms for ongoing education and support.

*“We conduct only an annual training about how to weigh and measure babies and children … Some of the [A&T-] trained staff have retired and their replacements have not been trained due to budget limitations. The district has no budget for training and the province does not conduct it.”* (Vietnam, female, sub-national KII)

*“Now refresher trainings include all issues, including essential health care, nutrition, tuberculosis … whereas during [A&T], we conducted a full-day refresher training, from 9 am until 4 pm, every quarter during the year, only about IYCF counseling, demonstration, problems they faced during IYCF counseling and their solutions*” (Bangladesh, male, sub-national KII)

We also investigated the persistence of health worker capacity to implement the activities post-A&T: knowledge, self-efficacy, and job satisfaction. Despite reductions in training, in both countries IYCF knowledge scores remained significantly higher among health workers in intervention areas versus workers in comparison areas (on average 11 and 14 percentage points in adjusted models, in Vietnam and Bangladesh respectively) (Table 3).

**Table 3:** Health worker IYCF knowledge scores

	Intervention area	Comparison area	Difference in score points (intervention vs. comparison)^1^ [SE]
**Bangladesh**	***n*** **= 300**	**n = 300**	
Health worker IYCF knowledge score	Average: 88.9% Range: 47.4–100%	Average: 70.7% Range: 42.1–100%	13.51*** points [0.73]
**Vietnam**	***n*** **= 44**	***n*** **= 24**	
Health worker IYCF knowledge score	Average: 83.0% Range: 52.6–100%	Average: 68.0% Range: 52.6–89.5%	10.98*** points [3.31]

*SE: standard error*

*IYCF: infant and young child feeding*

Level of significance: * < 0.05, ** < 0.01, *** < 0.001

^1^ *Linear regression model (OLS) that includes fixed effects (division-level in Bangladesh, province-level in Vietnam) and covariates: worker age, years of experience, years of education. Standard errors in brackets (clustered at lowest sampling unit level in Bangladesh)*

However, these high knowledge scores did not translate into better counseling: when counseling sessions were observed, workers in intervention areas of both countries performed on average only 36–46% of all recommended activities (Appendix 5). Stakeholders likewise discussed the deteriorating quality of counseling activities post-A&T, during interviews and focus groups in both countries. Time constraints were commonly cited as a barrier.

“*It is a time-consuming process. Now, instead of half an hour, they only spend 10 minutes. So the practice is not done properly. Sometimes they use mobile phones for counseling on IYCF, rather than directly demonstrating*.” (Bangladesh, male, FGD)

“*We do not follow the 8 topics as in the protocol, since it will take too long and women cannot stay so long. They ask for what they need, and we answer. We can also add a little information about nutrition. If we followed the protocol, it would be very difficult to conduct counseling*” (Vietnam, female, FGD)

Changes to monitoring, such as cuts in supportive supervision and quality improvement feedback mechanisms, were also noted as a factor affecting counseling. Stakeholders were very enthusiastic about monitoring during A&T, but cited subsequent deterioration in the degree of specificity (i.e., broadening the focus from IYCF to general nutrition indicators) and in feedback mechanisms.

“*During [A&T], the monitoring system was effective: one monitor worked for two upazilas [sub-districts], and monitored field activities by rotation, collecting monthly progress reports, and randomly observing workers’ IYCF counseling activities. Later we discussed their findings and feedback during the refresher trainings. But post-[A&T], these activities are being supervised by a general monitoring team focused on overall health and nutrition activities, not specific to IYCF*” (Bangladesh, male, sub-national KII)

“*No one supervises the counseling session anymore or reviews the monitoring book of client counseling*” (Vietnam, female, sub-national KII)

Health workers in intervention areas of Bangladesh nonetheless reported significantly higher job satisfaction (86%) and self-efficacy (93%) scores than workers in comparison areas (scores of 83 and 85% respectively) (Table 4). These relationships remained significant in multivariable models, with health workers in intervention areas scoring 7.7 points higher self-efficacy scores and 3.5 points higher job satisfaction scores. No such significant differences were seen among health workers in intervention versus comparison areas of Vietnam.

**Table 4:** Self-efficacy and job satisfaction of health workers

	Intervention area	Comparison area	Difference in rating points (intervention vs. comparison)^**1**^ [SE]
**Bangladesh**	**n = 300**	**n = 300**	
Self-efficacy rating	Average: 92.8% Range: 66.7–100%	Average: 85.2% Range: 33.3–100%	7.70*** [0.88]
Job satisfaction rating	Average: 85.9% Range: 50–100%	Average: 82.9% Range: 32.5–100%	3.54** [1.13]
**Vietnam**	**n = 44**	**n = 24**	
Self-efficacy rating	Average: 84.9% Range: 60–100%	Average: 80.3% Range: 60–100%	2.91 [3.80]
Job satisfaction rating	Average: 77.1% Range: 50–95%	Average: 75.7% Range: 57.5–97.5%	−1.36 [3.39]

*SE: Standard error*

Level of significance: * < 0.05, ** < 0.01, *** < 0.001

^*1*^*Linear regression model (OLS) that includes fixed effects (division-level in Bangladesh, province-level in Vietnam) and covariates: worker age, years of experience, years of education. Standard errors in brackets (clustered at lowest sampling unit level in Bangladesh)*

### What were stakeholders’ impressions about *determinants* of the sustainability of the A&T model and activities?

Qualitative data provided insights into specific determinants of sustainability highlighted in the study’s conceptual framework. Respondents from both countries felt that the government should take a leadership role for sustaining IYCF activities; and stakeholders in Vietnam spoke about the importance of involving all levels of the government, noting the important role of the national government in promoting these efforts.

“*Government should be the first changer in mainstreaming or implementing the IYCF activities nationwide*” (Bangladesh, female, national KII)

“*In order to integrate the program into the existing system in a sustainable manner, it should be start with the national level. If not, it is difficult for the provincial level to implement and integrate without guidance from the national level*” (Vietnam, female, sub-national KII)

“*Alive & Thrive was a comprehensive approach with the engagement of government stakeholders, which made the initiative acceptable by all levels of stakeholders.*” (Bangladesh, male, national KII)

There were, however, concerns about the requisite financial resources for sustaining program activities.

“*The modality was good, but who has so much money?... If anyone wants to replicate this in another country without funding from Bill and Melinda Gates Foundation, nobody will able to do it in the same way*” (Bangladesh, female, national KII)

*“During the [A&T] dissemination workshop, they presented the scaling-up plan and the proposed budget … But we waited and waited, and no funding came.”* (Vietnam, male, sub-national KII)

Stakeholders in Vietnam were concerned about the sustainability implications of unreliable public sector fund allocation for IYCF, increasing privatization of the health sector, and of omitting IYCF counseling from the national health insurance program.

“*I am concerned about how we can continue to provide counseling. In my opinion, if the Ministry of Health advised the government that nutrition and nutrition counseling should be paid by the health insurance, sustainability will be feasible.*” (Vietnam, male, sub-national KII)

*“In the last two years, the National Nutrition Program budget has been cut significantly. In some provinces, the budget is only 10% of what was planned.”* (Vietnam, female, national KII)

Partnership, including with civil society, and wide stakeholder buy-in were seen as important for sustainability – but stakeholders in both countries worried such efforts struggled without A&T as a coalescing force.

*“[The National Institute of Nutrition] is lonely now in the fight … A&T was independent so could link multi-disciplinary partners well.”* (Vietnam, female, national KII)

Turnover of human resources, at both leadership and implementation levels, was mentioned as a major sustainability challenge in both countries.

“*Staff were changed and retired, but the program did not monitor this closely to train new staff*” (Vietnam, female, sub-national KII)

“*If one [worker] dropped out, we had to distribute her registers among 8-10 others to manage the regular household visit. This increasing workload causes more dropouts.*” (Bangladesh, female, national KII)

Some respondents also mentioned low IYCF knowledge among new leadership as a challenge – and there were concerns about the continuity and long-term commitment of champions and loss of institutional knowledge due to high turnover.

“*Sometimes the government appoints a senior person in such designation which requires long experience in the field of nutrition, whereas the person might not have any exposure of nutrition related works*” (Bangladesh, male, national KII)

“*The head of [facility A], an active and very good manager of the A&T model, retired – and her replacement does not know much about the activities. In [facility B], the head and the nurse who were trained by A&T have been moved to another facility, and the newcomers do not know about A&T*” (Vietnam, female, sub-national KII)

The multi-pronged design of A&T was seen as key to its success, but stakeholders were concerned about sustaining this – particularly because the varied activities require expertise, support and leadership from different implementing agencies. For example, stakeholders felt as though different groups would best implement interpersonal communication versus mass media efforts, and were concerned that this fragmentation may add coordination challenges and undermine sustainability.

Stakeholders also discussed the importance of adaptations – including to training and interpersonal communication IYCF counseling materials – which have occurred during and subsequent to the program period. This flexibility was seen as a key strength of the model and important for integration and sustainability. A more problematic adaptation has been a broadening of scope since the end of A&T: improvements on specific IYCF indicators was seen as evidence of success in both countries, but stakeholders felt as though these have been diluted, and that stakeholders are less familiar with the specific goals. In both countries, this shift was attributed to competing priorities and the introduction of new programs and initiatives.

A summary of qualitative findings in each country, mapped to the theoretical constructs of sustainability, is presented in Table 5.

**Table 5:** Perceived determinants of sustainability (factors that may affect sustainability of A&T programs post-Phase 1) according to key stakeholders

Factor	Bangladesh	Vietnam
Program/project-specific factors		
Clear goal(s)	Clear goals of A&T have been diffused after Phase 1	Less awareness around specific goals post-Phase 1; urgent priorities (e.g., emergency situations) now distract focus
Perceived effectiveness	Phase 1 activities were highly effective but not sustained	
Financing	Challenging to raise funds after Phase 1; new donor priorities shape focus of activities; reduced incentives for health workers affect service quality	Reduced IYCF funding which affects post-Phase 1 activities; concerns about national and sub-national budgets; workers are seeking alternative sources of income (e.g., selling supplements, charging for services)
Training	Refresher trainings and supportive supervision were seen as essential to Phase 1 success, but have been eliminated/reduced, which affects service quality	
Evaluation/ assessment	Rigor & focus of M&E has declined post-Phase 1	
Leadership	Government should lead for sustainability	Government leadership seen as critical during and after Phase 1 (national and local); weaker convening power post-Phase 1
Organizational factors		
Local-level modifiability	Small adaptations to communication and training materials post-Phase 1; health workers modified counseling materials & content to reflect available resources and client preferences	
Initiative−/Donor- client/−community interactions	(Did not emerge as a theme)	
Project champion	High turnover of government officials in leadership positions	Champion role needs to expand/shift agencies post-Phase 1 which is challenging
Integration	Integration of IYCF programs into existing programs seen as essential; but disagreements about whether a dedicated workforce is needed	Advantages and challenges of franchises versus routine services (including competing demands on health workers); integration needs money and commitment from all levels of government
Institutional strength/capacities	Availability of skilled personnel, both frontline workers and leadership, is an ongoing challenge; media campaign capacity is weak post-Phase 1	High staff turnover affects sustainability; hard to ensure ongoing quality of community support group leaders
Contextual factors		
Concurrent projects/ activities	Other IYCF programs have shifted priorities (from government to workers) post-Phase 1	Many IYCF programs and donors, but overall perceived decline in priority
Community characteristics	Certain messages (handwashing) require repeated messaging which is challenging to sustain	Hard-to-reach communities (working mothers, geographic terrain) affects ongoing implementation; partnerships can help
Political, economic and cultural characteristics	Certain cultural beliefs perceived as continuing to inhibit IYCF behavior change	Lack of IYCF and antenatal services within national health insurance scheme may affect long-term sustainability (both financial support and demand for services), amplified by increasing female labor force participation (limited time to access care)

*A&T: Alive & Thrive*

*IYCF: infant and young child feeding*

*M&E: monitoring and evaluation*

## Discussion

This sustainability evaluation examined ongoing implementation of Alive & Thrive program activities in Bangladesh and Vietnam more than two years after external funding ceased. We found that many activities specifically aimed at promoting IYCF counseling with caregivers had continued in each country, thus expanding the reach of IYCF counseling services beyond the original program beneficiaries. In Bangladesh, the government successfully incorporated IYCF counseling and household visits into routine health programs. In Vietnam, although the national government was less heavily involved in the program, several provincial governments expanded the IYCF social franchises into new areas, and IYCF counseling is now included in the standard postnatal package for mothers.

The results also demonstrate the significant enduring capacity of health workers based on increases in intervention sites compared to comparison sites for health worker IYCF knowledge, self-efficacy, and job satisfaction. These knowledge scores were very similar to the results found in the A&T endline evaluations (see Appendix 6) [9] suggesting little “voltage drop” in this outcome over the extended time period [37]. However, in both countries, IYCF counseling activities in the intervention areas appeared to have worsened in quality since the end of the program. For example, during direct observation, many specific recommended IYCF counseling activities were no longer provided. Similarly, a systematic review about program sustainability found that fewer than half of providers, when observed, continued to implement the intervention over time at high levels of fidelity [20].

We also sought to understand why persisting higher knowledge did not necessarily translate into persisting higher-quality counseling as observed in the quantitative results, i.e. observed “know-do gaps” -- which are well-documented in the broader public health literature [45–48] but have not been previously applied in the context of sustainability. A major factor that emerged from the qualitative data was the introduction of new competing incentives (e.g., profits from selling commodities or services) and, in Bangladesh, the suspension of A&T-associated incentives. Earlier studies of A&T implementation similarly identified performance-based incentives and training/monitoring as instrumental for promoting IYCF counseling fidelity among frontline workers [35, 49]. Other studies have also suggested that incentive payments affect motivation and performance [50–52]. Although there may be an unavoidable tension between fidelity and adaptation when considering sustainability [20], researchers should assess the potential impact of different types of incentives (such as titles or training credits) to ensure sustained outcomes over time. Mixed methods studies like this one offer unique opportunities for exploring such mechanisms.

Institutionalization is generally viewed as an enabler or a necessary component of sustainability, or even as synonymous with “sustainability” itself although there is increasing push-back against this conceptualization [39, 53] – but these findings suggest it may have contributed to the decline of some program activities and monitoring. Stakeholders spoke about the importance of an IYCF-focused monitoring system with rapid feedback mechanisms during A&T, and described the detrimental effects of shifting to routine monitoring through existing systems. Previous studies have pointed to the importance of monitoring and feedback for program implementation [54], but fewer have examined it as a driver of sustainability [20]. Although “program drift” may be advisable as programs adapt to real-world long-term implementation [37, 39], partners may wish to consider counteracting the possible negative consequences of institutionalization, including attempting to maintain program integrity through earmarked budgets, dedicated program staffing, on-going training (particularly to replace rotating staff), topic−/activity-specific mentorship and supportive supervision activities, and detailed indicators in the monitoring system. A broader and more dynamic view that sees sustainability as independent of organizational structures -- and not necessarily dependent on or equivalent to institutionalization -- may be a more instructive approach for policymakers and planners [19, 39].

The multi-pronged design of A&T has been widely praised as being key to its success, however, it proved to be challenging to sustain, an important lesson for other at-scale complex programs. For example, this study found that many community-based and regional health education and advocacy activities ceased after the end of the project period (including broad mass media messaging and social mobilization efforts) despite stakeholders’ perception that these were impactful during the initial project period. Consistent with other sustainability frameworks that point to the importance of a “champion” with a strong commitment to the program [18, 26, 55], we found that champions were important during adoption and implementation – but these champions did not extend beyond the program period, particularly in the context of institutionalization. It may be important to identify and cultivate champions who can be supported beyond the life of a project, to either continue in this role or mentor new “sustainability champions” with skills in advocacy and problem solving.

This study also identified declines in IYCF-specific budgets in both countries post-A&T. Both public and private sources of financing were perceived to have limitations: allocations of public sector funds can be unreliable, while private sector support may come with new restrictions or different priorities. These funding challenges have caused the sunsetting of entire program categories, alteration (and in some cases narrowing) of target geographies and/or populations, and cuts to activities including health worker IYCF counseling training, and monitoring and evaluation. An examination of changes in funding allocation and sources (private and public) is important for understanding program sustainability [20] as different funding sources present unique benefits and challenges [56]. This model provided IYCF services free of charge; other strategies might seek financing options to establish a more sustainable long-term source of revenue [35].

This study adds to a growing global literature about sustainability [17, 25, 26, 57–60] with a unique mixed methods study design. We incorporated a strong theoretical foundation, and leveraged a quasi-experimental design to compare measurable and observable outcomes in intervention versus comparison areas over a long post-implementation period – a rare opportunity in implementation research [25, 39] particularly in low-resource settings [41]. Measuring the evolving and complex concept of sustainability remains a challenge. By using qualitative methods to deepen our understanding of how multiple determinants may have affected the quantitatively identified outcomes, we made progress in operationalizing and disentangling the mechanisms of sustainability, yet could not fully capture the dynamic complexity inherent in a sustainable program [37].

Some additional limitations should also be noted. First, these methods were not designed to assess causality. The study was also not designed or powered to compare findings across the two study countries. Additionally, the health worker sample in each country is very different – both in terms of background/cadre as well as sample size (due to sample selection strategies which differed by country to reflect A&T program structure). On the latter point, it should likewise be noted that the relatively small number of health workers surveyed in Vietnam may have limited our ability to find statistically significant differences between intervention and comparison area groups. Second, response bias may have affected some of these results: health workers may over- or under-state job satisfaction or self-efficacy, and there may have been social desirability bias for qualitative respondents. We tried to minimize these biases by using data collection teams unaffiliated with A&T program implementation, but it impossible to eliminate all such reporting bias. Similarly, the service observation scores may be biased upward due to a Hawthorne effect, or downward if the research assistants faced challenges in accurately documenting all aspects of an IYCF counseling session. Finally, all interviews and focus groups were conducted in each country’s local language, and full translations were beyond the scope of this project so separate research teams conducted the qualitative analyses. We implemented multiple quality assurance mechanisms to minimize potential bias by extensively discussing the codebooks and preliminary results, as well as assessing inter-rater reliability on a subset of translated transcripts.

## Conclusions

This research examined the sustainability of a comprehensive set of interventions that were designed and implemented with an explicit sustainability plan. The results suggest that while some program activities have been institutionalized and continue to be implemented after the end of the project, there have been substantial attenuations in implementation of certain activities (while other activities have ceased altogether). Despite this, some program benefits persist including strengthened capacity of health workers in intervention areas. The findings of this study also show that, while many earlier lessons from the field of sustainability research are still valid, there are some nuances that require additional consideration. For example, earlier literature emphasizes the importance of integration of activities into existing institutions. This study found that integration, if not accompanied by mechanisms to maintain program or activity fidelity, may lead to deterioration. The potential benefits of integration should therefore be considered within this context, and the trade-offs between institutionalization and the dimensions of sustainability may vary across activities and contexts. Furthermore, neither robust results nor thorough integration guarantees a continuation of funding, and the search for future funding introduces new challenges for sustainability. It was also found that champions were important during implementation, but strategies should be developed for identifying, cultivating and maintaining champions despite high turnover to ensure a program’s sustainability. Future sustainability studies should explore whether programs are diluted, subsumed or transformed due to adaptations such as discontinuation of activities over time [20]. It is likely that further research into the sustainability of innovative projects will produce new and important recommendations for effective sustainability strategies.

## Supplementary information

**Additional file 1:** Appendices.

## Data Availability

The datasets used and analyzed during the current study are available from the corresponding author on reasonable request.

## Abbreviations

A&T: Alive & Thrive initiative
FGD: Focus group discussions
IYCF: Infant and young child feeding
KII: Key informant interviews
MTBT: Mặt Trời Bé Thơ
