# Supplementary material for: Can complex programs be sustained? A mixed methods sustainability evaluation of a national infant and young child feeding program in Bangladesh and Vietnam
Source: BMC Public Health. 2020 Sep 4;20:1361. doi: 10.1186/s12889-020-09438-2 (PMC7487916; doi:10.1186/s12889-020-09438-2)
Supplement: Supplementary file 1 — Additional file 1: Appendices. [file 12889_2020_9438_MOESM1_ESM.docx]

**Appendices**

1. Activities associated with sustainability strategies in each study country
2. Comparing frameworks of sustainability determinants
3. Sample selection procedure
4. Descriptive information on study participants
5. Service observation scores
6. Comparison of sustainability dimensions to endline evaluation values
7. **Activities associated with sustainability strategies in each study country**

| **Strategy** | **Bangladesh Examples** | **Vietnam Examples** |
| --- | --- | --- |
| 1. Strengthen the enabling environment through advocacy for policy change | - Increased funding by raising awareness among policymakers - Incorporated IYCF training into national medical and nursing curricula | - Extended maternity leave from 4 to 6 months - Strengthened law banning advertising of breast milk substitutes |
| 1. Explore sources of continued financial investment for IYCF services | - Identified funding in national budget for scale up of IYCF services - Sought funding from other development partner by assisting with proposal writing and with presenting evidence of program impact | - Encouraged People’s Councils to establish user fees for IYCF services - Phased out A&T budget and increased contribution of provinces - Worked towards policy change for IYCF services to be covered by government health insurance |
| 1. Encourage adoption of key components of A&T model by governmental and nongovernmental institutions | - Built strong alliance with and gave ownership of program components to Institute of Public Health Nutrition - Provided new stakeholders with technical support, research results, advocacy tools, seed funding, and adaptation of IEC materials | - Established social franchises in government health facilities - Collaborated with National Institute of Nutrition as a co-franchisor - Developed hand-over plans to give ownership of programs to provincial partners |
| 1. Build capacity and disseminate tools for sustaining and expanding the A&T model | - Developed training materials, job aids, and communication materials for program managers and IYCF service providers - Trained personnel to design, implement, monitor, and evaluate programs - Built expertise in local institutions to generate continuing evidence on program effectiveness - Embedded IYCF in longer term capacity building of health providers and adolescents | - Developed training materials, job aids, and communication materials for program managers and IYCF service providers - Trained personnel to design, implement, monitor, and evaluate programs - Engaged with institutions to ensure they have the understanding and capacity to sustain the programs - Sought to maintain the brand integrity of the social franchises |
| 1. Increase public awareness of IYCF services to generate community demand | - Conducted an ongoing mass media campaign - Engaged with community leaders | - Conducted an ongoing mass media campaign - Engaged with community leaders |

1. **Comparing frameworks of sustainability determinants**

| **Bossert (1, 2)** | **Bao (3)** | **Gotsadze (4)** | **Hailemariam (5)** | **Iwelunmor (6)** | **Lee (7)** | **Shediac-Rizkallah (8)** | **Shelton (9)** | **Stirman (10)** |
| --- | --- | --- | --- | --- | --- | --- | --- | --- |
| **Clear goal(s)** | Agreement on objectives |  | Fit | Fit |  |  |  | Fit |
| **Perceived effectiveness** | Seen as valuable |  | Effectiveness, benefit | Perceived benefits/risk | Program effectiveness | Project effectiveness | Benefits/ need | Effectiveness/ benefit |
| **Financing** | Adequate financing with management systems | Financial resources | Funding | Financial infrastructure | Funding source | Project financing | Funding environment; Funding/ resources | Funding |
| **Training** | Training activities |  | Training and education | Staffing and training |  | Training | Technical assistance; Capacity building | Training and education |
| **Evaluation/assessment** | M&E | Information systems; accountability | Evaluation and feedback | Assessment and learning |  |  |  | Evaluation and feedback |
| **Leadership** | Transparent government leadership |  | Organizational leadership | Leadership; Supervision | Political commitment | Leadership |  | Leadership |
| **Local-level modifiability** | Match of problem and program |  | Adaptation/alignment; ability to modify | Local adaptation | Negotiation; Communication | Project negotiation process | Adaptability | Ability to be modified; Adaptation/ alignment |
| **Initiative-/Donor- client/-community interactions** | Commitment from political levels |  | Engagement/relationship building; Shared decision making; Community support, involvement | Community dialogue, ownership and engagement; Stakeholder involvement | Community participation | Community participation | Community ownership; Partnership/ engagement | Community/stakeholder support/ involvement; Shared decision-making |
| **Project champion** |  |  | Internal/external champions | Project champion |  | Project champion |  | Champions (internal or external) |
| **Integration** | Activities integrated into routine operations |  | Integration of rules and policies |  | Institutionalization | Integrating with existing programs/ services |  | Integration of rules/ policies |
| **Institutional strength/capacities** | Technical, managerial and financial capacity | Human resources; organizational capacity | Workforce; Resources; Ongoing support | Local resources; Staffing and training | Capacity building | Institutional strength | Capacity | Workforce (staffing, attributes); Resources; Ongoing support |
| **Concurrent projects/ activities** | Alignment with other activities |  | Collaboration, partnership; Navigating competing demands |  | Linkage, connectedness |  | Mission | Collaboration/ partnership; Navigating competing demands |
| **Community characteristics** | Community support |  | Setting characteristics (structures, policies) | Norms and values |  |  | Values, priorities, needs | Culture; Setting characteristics |
| **Political, economic and cultural characteristics** | Political environment and economic situation | Political and economic environment | System/policy change | Policies/politics | Socio-culture | Socioeconomic and political considerations | Sociopolitical context; Population characteristics | Climate; System/ policy change |
| (Other) |  |  | Organizational climate; Planning; Organizational culture; Ability to maintain integrity |  | Transition | Project duration; Project type | Cost; Provider characteristics; Implementation skills/expertise | Ability to maintain fidelity/ integrity; Engagement/ relationship building; Planning |

1. **Sample selection procedure**

1. **Descriptive information on study participants**

Characteristics of study population: Health workers

|  | **Bangladesh, health workers** | | **Vietnam, health workers** | |
| --- | --- | --- | --- | --- |
|  | **Intervention Area**  **(n=300)** | **Comparison Area**  **(n=300)** | **Intervention Area**  **(n=44)** | **Comparison Area**  **(n=24)** |
| **Age (years), mean (± SD)** | 39.9 (± 10.8) | 40.3 (± 10.9) | 40.6 (± 7.6) | 43.3 (± 9.1) |
| **Position in health sector, % (n)** | | | | |
| SS/PS | 61.0% (183) | 78.0% (234) | N/A | N/A |
| SK/PK | 39.0% (117) | 22.0% (66) | N/A | N/A |
| Doctor | N/A | N/A | 20.5% (9) | 20.8% (5) |
| Nurse | N/A | N/A | 11.4% (5) | 0 |
| Midwife | N/A | N/A | 48.8% (21) | 22.2% (6) |
| Pharmacist | N/A | N/A | 0 | 1.3% (1) |
| Physician’s assistant | N/A | N/A | 20.5% (9) | 22.2% (6) |
| **Years of schooling, mean (± SD)** | 7.2 (± 6.7) | 6.8 (± 6.4) | n/a | n/a |
| **Years of experience in role, mean (± SD)** | 7.6***(± 5.0) | 9.9 (± 6.0) | N/A | N/A |
| **Years of experience in health sector, mean (± SD)** | N/A | N/A | 17.4 (± 7.8) | 18.8 (± 8.4) |
| **Years of experience in IYCF, mean (± SD)** | N/A | N/A | 9.9* (± 7.2) | 14.0 (± 7.8) |
| *Level of significance of t-test of difference between intervention and comparison areas per indicator* < 0.05, ** < 0.01, *** < 0.001*  SS: Shasthya Shebika; PS: Pushti Shebika; SK: Shasthya Kormi; PK: Pushti Kormi | | | | |

Characteristics of study population: Qualitative respondents

|  | | |
| --- | --- | --- |
|  | **Bangladesh** | **Vietnam** |
| National key informant interviews | n=14 | n=16 |
| Sex: Male | 7 | 8 |
| Female | 7 | 8 |
| Sector: Public (domestic) | 2 | 11 |
| Donor agency, development partner | 3 | 2 |
| Private sector, not-for-profit | 7 | 3 |
| Private sector, for-profit | 2 | 0 |
| Sub-national key informant interviews | n=10 | n=105 |
| Sex: Male | 8 | 36 |
| Female | 2 | 69 |
| Sector: Public (domestic) | 2 | 105 |
| Donor agency, development partner | 1 | 0 |
| Private sector, not-for-profit | 7 | 0 |
| Level: Province | 0 | 26 |
| District | 6 | 26 |
| Sub-district | 4 | 53 |
| Focus group participants | n=43 | n=30 |
| Sex: Male | 32 | Not available |
| Female | 11 | Not available |

1. Service observation scores: Average number of IYCF activities (total score, IYCF technical items score, and service score) performed by health workers, on 0-100 scale, in Bangladesh (left) (n=242) and Vietnam (right) (n=27)

**
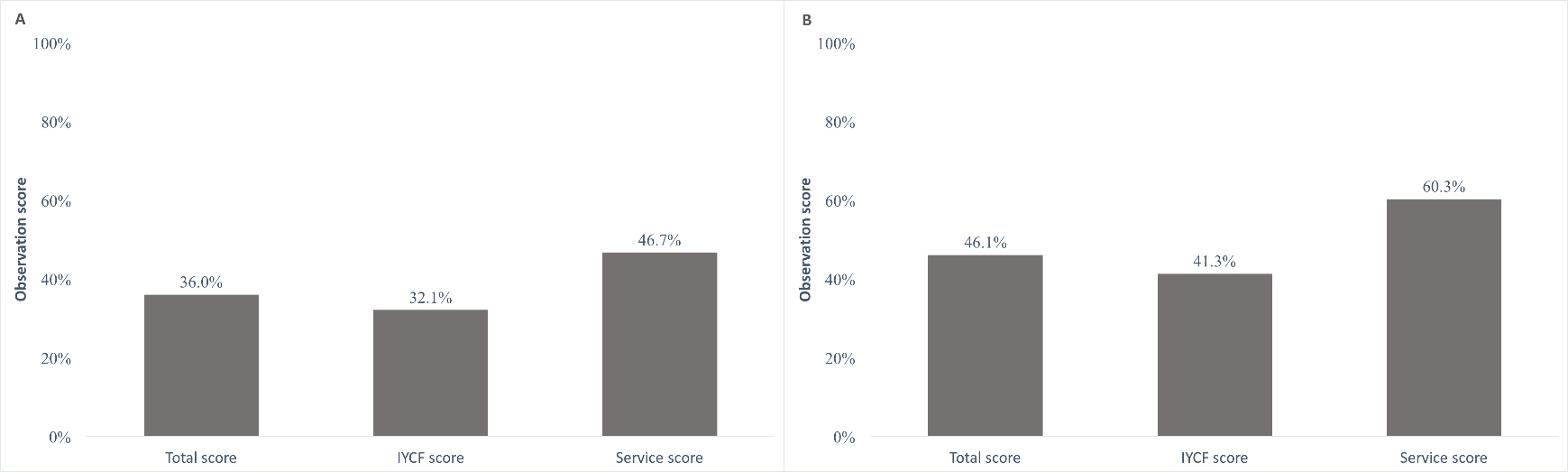
**

1. **Comparison of sustainability evaluation outcomes to endline evaluation values**

|  | Sustainability study measure: Intervention areas | Endline study measure: Intervention areas | Sustainability study measure: Comparison areas | Endline study measure: Comparison areas |
| --- | --- | --- | --- | --- |
| **Early initiation of breastfeeding** | | | | |
| Vietnam | 54.3 [46.6, 61.8] | 53.2 | 48.1 [40.6, 55.6] | 40.6 |
| Bangladesh | 87.3 [78.0, 93.8] | 94.3 | 65.0 [53.5, 75.0] | 75.7 |
| **Exclusive breastfeeding under 6 months** | | | | |
| Vietnam | 55.0 [38.5, 70.7] | 53.2 | 40.7 [22.4, 61.2] | 28.4 |
| Bangladesh | 90.0 [76.3, 97.2] | 94.3 | 55.0 [38.4, 70.7] | 53.5 |
| **Health worker breastfeeding knowledge** | | | | |
| Vietnam | 89.3 | 85.6 | 68.2 | 68.9 |
| Bangladesh | 88.2 | 92.2 | 71.6 | 80.0 |
|  |  |  |  |  |

Endline study data published at: Menon P, Nguyen PH, Saha KK, Khaled A, Kennedy A, Tran LM, et al. Impacts on Breastfeeding Practices of At-Scale Strategies That Combine Intensive Interpersonal Counseling, Mass Media, and Community Mobilization: Results of Cluster-Randomized Program Evaluations in Bangladesh and Viet Nam. PLoS Med. 2016;13(10):e1002159
